# Knowledge of health professional students on waterpipe tobacco smoking: curricula implications

**DOI:** 10.1186/s12909-018-1406-9

**Published:** 2018-12-07

**Authors:** Randah Ribhi Hamadeh, Jamil Ahmed, Ghufran Ahmed Jassim, Sayed Mahmood Alqallaf, Khaldoon Al-Roomi

**Affiliations:** 1 Department of Family and Community Medicine, College of Medicine and Medical Sciences Arabian Gulf University, P O Box 26671, Manama, Bahrain; 2 Department of Family Medicine, Royal College of Surgeons Ireland–Bahrain, Busaiteen, Bahrain; 3 College of Health Sciences, University of Bahrain, Zallaq, Bahrain

**Keywords:** Waterpipe tobacco smoking, Educational intervention, Health professionals, Knowledge, Health effects, Medical curriculum, Medical education

## Abstract

**Background:**

Tobacco prevention research traditionally focuses upon cigarette smoking, but there is also a need to implement and evaluate the usefulness of waterpipe tobacco smoking (WTS) interventions since it is considered less harmful than cigarettes. This study aimed to assess the impact of an educational intervention on WTS knowledge of health professional students in three academic health institutions in Bahrain.

**Methods:**

A quasi-experimental design was used to include medical students from the Arabian Gulf University, medical and nursing students from the Royal College of Surgeons in Ireland-Bahrain and nursing students from the University of Bahrain. Two hundred fifty students participated in the three phases of the study during October 2015–June 2016 from an original sample of 335. The participants answered knowledge questions on WTS before and after an intervention, which included a lecture by an expert and a video on the awareness about the health hazards of WTS.

**Results:**

The mean age of starting cigarette and WTS was 16.8 ± 2.8 and 17.5 ± 1.7 years, respectively. The prevalence of ever smoking any type of tobacco among students was 22.4% (medical 25.8% and nursing 37.5%) and that of WTS, 17.7% (medical 20.0%, nursing 13.6%). The prevalence of current cigarette smoking was 9.6% among medical and nursing students combined with 10.3 and 8.5% for medical and nursing students, respectively. WTS was prevalent at a proportion of 6.8% among medical and nursing students combined with 6.5% in medical and 14.8% in nursing students. The university curriculum as the main source of knowledge on WTS increased from 14.2 to 33.3% after the intervention (*p* < 0.005). Knowledge about the hazards of WTS increased in 16 of the 20 statements. The difference in overall knowledge score was significant (*p* < 0.05) for nursing (77. 5 ± 1.5 vs 85.8 ± 2.2) compared to medical students (85.3 ± 1.0 vs 87.3 ± 0.9) after the intervention.

**Conclusions:**

Our educational intervention with health professional students improved their knowledge about the health effects of WTS. Medical and nursing institutions may consider using various methods such as informative videos and expert lectures to include in their teaching curricula as part of WTS prevention strategies.

## Background

Despite the significant gains by global tobacco control efforts since 1990, a quarter of the world’s population still smokes various forms of tobacco [1]. In 2015, the Global Burden of Disease Study ranked tobacco smoking among the top five risk factors responsible for disability-adjusted life years lost in 109 countries [1]. Even though the burden of cancer and other non-communicable diseases linked with tobacco use is shifting to developing countries [2], yet developed countries continue to bear this burden. In Bahrain, lung cancer is the leading type of cancer among males with an age-standardized incidence rate of 27.5/100, 000, and the third most common in females (10.5/100, 000) during 1998–2012 [3]. The country has over 364 deaths attributable to tobacco-related diseases annually [4]. The burden of tobacco-related mortality is considered critical given the small population size of the country. Bahrain had a population size of 1.316 million in 2014 which is projected to reach 1.592 million by 2020 [5]. There is a complete ban on tobacco advertisement and promotion with 18 years being the legal age for purchasing tobacco in the country [6]. The age-standardized estimated prevalence of any form of tobacco smoking in adults, 15 years and older, is 30.3% [7]. A higher prevalence of tobacco smoking among medical students in Bahrain was noted over time. It increased in males from 27.5% in 1993 to 35.2% in 2005 and in females from 2.3 to 7.0% during the same period [8].

There has been an alarming increase in waterpipe tobacco smoking (WTS) among youth in the Eastern Mediterranean Region as it is increasingly considered as an acceptable alternative to cigarettes [9]. Among those 20–64 years old, 10.8% of males and 6.2% of females are waterpipe smokers in Bahrain [10]. The trend of WTS is also critically high among healthcare professionals. About 12% male and 2% of female primary healthcare physicians in the country currently smoke waterpipe [11]. Addiction to WTS in later years of life starts much earlier in healthcare workers and WTS prevalence in this population has similar patterns and trend, as is in the general population. For instance, only 8.8% male and none of the female medical students smoked waterpipe in 1993. There was a sharp increase in their use of WTS in the next decade, leading to a WTS prevalence of 31.3% in males and 6.1% in females in 2005 (8). This has been attributable to the fact that training of Bahraini medical students and physicians lacks in the area of tobacco dependence prevention and cessation techniques [8, 12].

The health hazards of WTS compared to cigarette smoking are underestimated by university students as well as the general population [13]. Their lack of knowledge about WTS is mainly because of the unavailability of information and misconceptions about WTS [14]. WTS is clearly associated with adverse cellular and genetic changes much like those caused by cigarette smoking. Even light (< 5 times /week) WTS is associated with moderate symptoms of cough and sputum production and later, with more advanced complications of the respiratory tract [15]. A recent case series study suggests that WTS is linked to acute carbon monoxide poisoning in young adults, to the extent of requiring emergency room visits [16]. Toxic effects of carbon monoxide may not be limited to waterpipe tobacco smokers but can also affect non-smokers from second-hand smoke [17]. Moreover, studies on the toxic contents and health consequences of WTS have identified around 300 health hazardous chemical compounds associated with lung, oral and urinary bladder cancers; and chronic obstructive pulmonary disease [18]. Not surprisingly, experts have called for evaluating the health messages and interventions for the prevention of WTS to create a powerful effect as has been for cigarettes [19]. Therefore, educating future physicians and nurses on the hazards of WTS could be an effective means of controlling WTS because they would lead the WTS cessation interventions in healthcare settings. It is imperative that they, as role models, avoid using any form of tobacco. Physicians are also expected to inquire from their patients about tobacco use to help them quit. A study from the United Kingdom reported that 69.5% of smoking cessation professionals never ask their patients about WTS use; even though three-quarters of the patients would like to have more information on WTS [20]. There are scarcely any studies from the Gulf Cooperation Council countries evaluating a WTS prevention strategy. Interventions to prevent and control tobacco smoking particularly WTS, have also been sparse [21, 22] and only a recent focus. The aim of the present study was to assess the impact of an educational intervention on the health professional students’ knowledge about WTS and the health effects associated with it.

## Methods

Bahrain has two medical and two nursing schools. This was a quasi-experimental study and participants included medical students of the College of Medicine and Medical Sciences, Arabian Gulf University (AGU) and the Royal College of Surgeons Ireland-Bahrain (RCSI Bahrain), nursing students from the College of Health Sciences (CHS), University of Bahrain and RCSI Bahrain. The total population of students studying in the three institutions was 2573 at the time of enrolment of participants. The numbers of medical students at AGU was 892 and RCSI Bahrain 750. There were 400 nursing students at RCSI Bahrain and 531 at CHS. It is worth noting that the majority of AGU students are Gulf Cooperation Council nationals, while those from RCSI Bahrain are international, mainly from Bahrain (39%), Kuwait (13%), Canada (10%), and 6.0% each from Jordan and the United States of America, while most CHS students are from Bahrain.

**Fig. 1:**
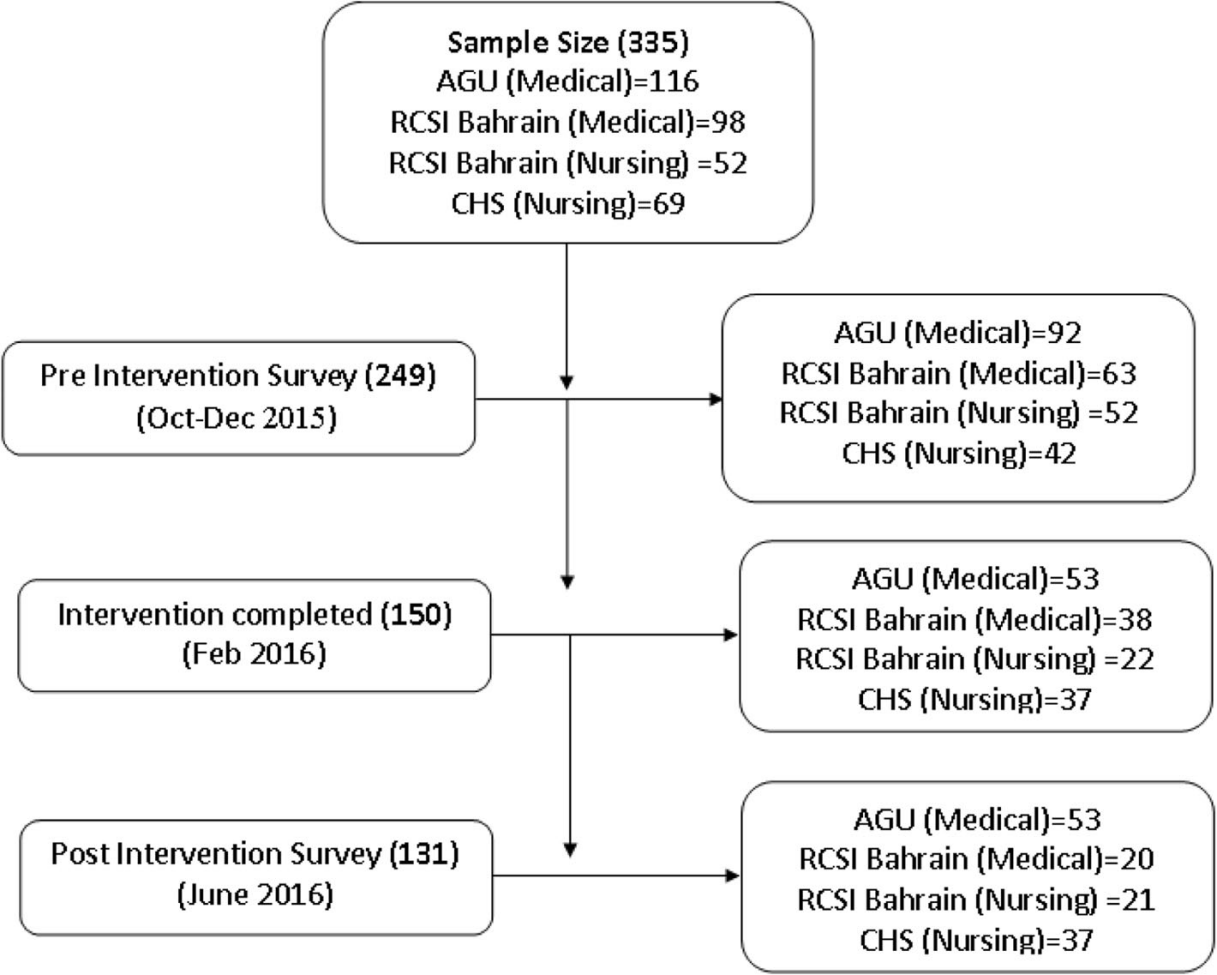
Project flow with phases of the study

A sample size of 335 students was obtained assuming that our intervention would result in a positive change in the 50% students’ WTS knowledge at 95% confidence level and a bound on the error of estimation of 0.05. A multistage stratified random sample was selected from the three institutions. Two hundred fourteen medical students (116 from AGU, 98 from RCSI Bahrain) and 121 nursing students (52 from RCSI Bahrain and 69 from CHS) were selected by simple random sampling from a list of students enrolled in each class for the academic year 2015–2016. We replaced students who refused to participate in the study by others from the original sampling frame (Fig. 1).

A focal person was identified from each school to distribute and collect the questionnaires from the participants. The questionnaires were self-administered, anonymous at pre-intervention and post-intervention phases and submitted after completion in sealed envelopes to the respective focal persons. The questionnaire included demographic and knowledge items on the health effects of WTS based on the knowledge statements developed by the Centers for Disease Control and Prevention, USA [23]. These 20 knowledge items comprised of true and false statements. For the assessment of WTS and cigarette smoking, standard core questions developed by Maziak et al [24] and the Global Health Professions Student Survey were also used [25]. The knowledge part of the questionnaire had a Cronbach’s Alpha of 0.75. The intervention included a 5-min video [26] and a lecture on the hazards of WTS by the principal investigator who is a tobacco research expert in Bahrain. Data for the pre-intervention phase were collected from October to December 2015, followed by the intervention in February 2016 and data collection for the post-intervention phase was done in June 2016.

Definitions used for WTS in our study included: Ever WTS: ever smoked one or two inhalations, current WTS: smoked waterpipe at least once in the previous month, former WTS: smoked waterpipe at least once a month for three consecutive months in the past, never WTS: never smoked waterpipe at all, and non-WTS: never smoked waterpipe or a former waterpipe smokers. For cigarette smoking definitions used in our study included: ever cigarette smoking: ever smoked cigarettes, even one or two puffs, current cigarette smoking: smoked at least 1 cigarette per day during the month preceding the survey, former cigarette smoking: quit cigarette smoking more than a month prior to completing the questionnaire and never cigarette smoking: never smoked cigarettes at all. A single knowledge score was computed from the knowledge variables on WTS and was compared for differences in levels of knowledge by cigarette smoking, WTS and all types of smoking status, gender and study track, nursing or medical. Twenty items related to knowledge about the health hazards of WTS had a total maximum score of 20, which was converted to “100” for convenience. Ethical approval was obtained from the ethical committees of all the three participating institutions. Participants gave written and informed consent before answering the questionnaires for both phases of the study.

## Results

Sixty percent of the students completed the three phases of this study. Most of the participating students were Gulf Cooperation Council nationals with females comprising 68.5% of the pre-intervention and 66.7% of the post-intervention sample. The mean age of students was 21.1 ± 1.6 years. The mean age of starting smoking was lower for cigarettes (16.8 ± 2.8 years) than WTS (17.5 ± 1.7 years) (*p* > 0.05). There were also no statistically significant differences between the study track and other characteristics of the participants after the intervention (Table 1).

**Table 1:** Characteristics of the participants in the study

		Pre Intervention		Post Intervention		*P* Value
		No.	%	No.	%	
Track	Medicine	155	62.2	73	55.7	0.240
	Nursing	94	37.8	58	44.3	
	Total	249	100.0	131	100.0	
Year	Year 1	71	28.5	23	17.8	0.299
	Year 2	51	20.5	25	19.4	
	Year 3	51	20.5	42	32.6	
	Year 4	42	16.9	25	19.4	
	Year 5	18	7.2	9	7.0	
	Year 6	16	6.4	5	3.9	
	Total	249	100.0	129	100.0	
Age (Years)	19	48	19.5	15	13.8	0.503
	20	42	17.1	24	22.0	
	21	58	23.6	30	27.5	
	22	64	26.0	28	25.7	
	23	34	13.8	12	11.0	
	Total	246	100.0	109	100.0	
Gender	Male	78	31.5	42	33.3	0.713
	Female	170	68.5	84	66.7	
	Total	248	100.0	126	100.0	
Nationality	GCC Citizen	219	88.0	118	90.0	0.518
	Others	30	12.0	13	10.0	
	Total	249	100.0	131	100.0	

**Fig. 2:**
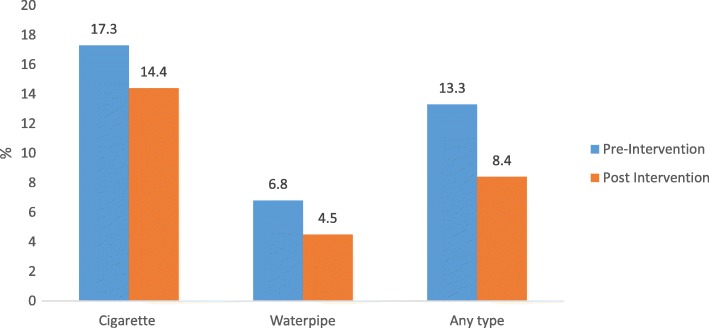
Current smoking of cigarettes, waterpipe and any type of tobacco during pre-intervention and post-intervention phases

Prevalence of ever and current use of waterpipe, cigarettes and smoking any type of tobacco decreased following the intervention (Fig. 2). Although current WTS prevalence decreased from 6.8 to 4.6% and cigarette smoking from 9.6 to 6.1% after the intervention, the differences were not statistically significant. The proportion of students who ever smoked, currently smoked, and smoked weekly and daily, and who shared the WTS session with others decreased. However, 83.3% participants after the intervention compared to 70.4% before, intended to quit WTS any time in the future. We also found that about two-thirds of the waterpipe smokers shared the mouthpiece with others during a smoking session.

The participants’ main source of knowledge on WTS was social media in both phases (44.5%, before and 42.4%, after the intervention). However, students’ sources of knowledge on WTS and cigarette smoking differed significantly. University curriculum as the main source of knowledge on hazards of WTS and cigarette smoking increased for cigarette smoking from 14.2 to 33.3% and for WTS from 28.4 to 46.1% after the intervention (*p* < 0.005). The proportion of students who correctly answered the knowledge statements increased in 16 and decreased in four of the 20 statements (Table 2).

**Table 2:** Knowledge about the health hazards of WTS Smoking: pre and post intervention

	Pre Intervention		Post Intervention		*P*-value
	No.	%	No.	%	
WTS is safe alternative to cigarette smoking (F)	233	93.6	122	92.8	0.918
The charcoal to heat tobacco raises health risks by producing high levels of CO (T)	226	90.8	114	87	0.259
After passing through water, smoke from WTS has lower levels of toxic agents (F)	188	75.8	114	87.0	0.01
WTS tobacco smoke contains toxic agents that cause lung cancer (T)	237	96.0	119	91.5	0.076
Tobacco juices from WTS do not increase the risk of developing oral cancer (F)	199	80.2	111	84.7	0.281
WTS tobacco and smoke contain toxic agents that cause coronary heart disease (T)	230	93.5	118	90.8	0.338
Infections may be spread by sharing WTS (T)	207	83.1	120	92.3	0.014
Pregnant women who smoked WTS do not have increased risk of having low birth weight babies (F)	216	86.7	114	87.0	0.94
Babies born to WTS smokers are at an increased risk of respiratory disease (T)	220	88.4	118	90.8	0.472
WTS smokers inhale lower amount of smoke than cigarette smokers (F)	211	85.1	114	87.0	0.607
WTS is less addictive than cigarette smoking (F)	132	53.2	94	72.3	< 0.001
The smoke of WTS is as toxic as that of cigarettes (T)	160	65.0	94	72.3	0.152
An hour-long WTS session has 200 puffs, and a cigarette has 20 puffs (T)	186	75.6	111	86.0	0.018
WTS has many of the same health risks of cigarettes (T)	219	88.3	113	86.3	0.565
Smoke inhaled during a typical WTS session is over 100 times than smoking a cigarette (T)	207	84.5	111	85.4	0.818
In comparison with cigarette smokers, WTS smokers are not at risk of decreased fertility (F)	190	76.9	112	86.2	0.033
In comparison with cigarette smokers, WTS smokers are not at risk of decreased lung function (F)	217	87.9	120	91.6	0.265
Smoke from *maasel* (herbalWTS) contains carbon monoxide (T)	201	81.4	117	90.0	0.028
Secondhand smoke from WTS is not a health risk for nonsmokers (F)	199	80.6	107	81.7	0.793
WTS among youth is decreasing worldwide (F)	187	75.4	104	79.4	0.382

*T* True, *F* False

Students’ correct responses for three statements improved significantly (*p* < 0.05) following the intervention. These statements were: “Infections may be spread by sharing WTS”, “An hour-long WTS session involves 200 puffs while smoking an average cigarette involves 20 puffs” and “Smoke from *maasel* (herbal WTS) contains carbon monoxide”. Similarly, students’ disagreement with three incorrect statements increased after the intervention (*p* = < 0.05). These statements included: “After passing through water, smoke from waterpipe has lower levels of toxic agents”, “waterpipe smoking is less addictive than cigarette smoking” and “in comparison with cigarette smokers, waterpipe smokers are not at risk of decreased fertility”. Further, there were not any statistically significant differences when the correct answers for the 20 knowledge items were compared across waterpipe and cigarette smokers.

Smokers, females and nursing students had better knowledge prior to and after the intervention (Table 3). The overall mean knowledge score increased from 82 to 86.7 after the intervention; however, it increased marginally for medical students but significantly for the nursing (*p* = 0.002). The score was higher for medical (85.4) than nursing (77.5) students at pre-intervention and reached 87.3 for medical and 85.8 for nursing students after the intervention. Knowledge about WTS improved across all academic years after the intervention. However, none of these differences was statistically significant.

**Table 3:** Students’ correct knowledge score by smoking status (all types), gender and track

Time	N	Mean	SD	SE	Mean Diff	95% C.I		*P*-Value
						Lower	Upper	
Pre Intervention								
Non Smoker	177	82.03	14.35	1.08	−1.44	−5.37	2.50	0.471
Smoker	49	83.47	11.65	1.66				
Post Intervention								
Non Smoker	97	85.67	17.18	1.74	−4.33	−8.78	0.12	0.057
Smoker	20	90.00	6.28	1.40				
Pre Intervention								
Male	70	82.36	14.06	1.68	0.28	−3.62	4.19	0.887
Female	159	82.08	13.71	1.09				
Post Intervention								
Male	39	88.21	13.35	2.14	2.76	−3.38	8.90	0.375
Female	79	85.44	16.93	1.90				
Pre Intervention								
Medicine	138	85.36	12.23	1.04	7.92	4.27	11.56	< 0.005
Nursing	92	77.45	14.63	1.53				
Post Intervention								
Medicine	69	87.32	15.89	1.91	1.55	−4.19	7.29	0.594
Nursing	52	85.77	15.67	2.17				

## Discussion

The present study assessed the impact of an educational intervention on the knowledge of medical and nursing students in Bahrain on WTS and its relationship with various health-related outcomes. The intervention, a combination of an informative video session followed by a tobacco research expert’s lecture, improved the students’ knowledge, and to some extent their smoking behaviour. Students’ knowledge about the health effects of WTS significantly improved and the overall mean knowledge score increased following the intervention. This change may be attributed to the intervention since no changes have occurred in the curriculum of these institutions during the study period that could have affected the students’ knowledge on tobacco health hazards. The fact that medical students were more knowledgeable than the nursing, as found in the pre-intervention survey, is most likely due to the higher coverage of the harmful effects of WTS in the medical curriculum. Further, the remarkable improvement in the knowledge of nursing students affirms the potentially important role that such an intervention can play in improving knowledge on the hazards of WTS. Although there are scarcely any similar studies, these results are consistent with studies that have repeatedly shown a positive impact of educational interventions on participants’ knowledge improvement [27]. A recent study showed that educational messages, even if delivered online, could have a positive impact on changing the population’s perceptions about WTS. However, the effectiveness of these messages in reducing the prevalence of WTS has not been encouraging [28].

Our pre-intervention survey showed that the university curriculum was not the main source of students’ knowledge about the hazards of smoking. The students’ knowledge of the hazards of cigarette smoking (28.4%) was twice that of WTS (14.2%). A possible explanation for this difference is that traditionally health professional students’ curricula in Bahrain have more emphasis on cigarette smoking. Thus, for future health professionals to become role models and help their patients quit tobacco smoking, they require a curriculum that adequately covers topics about the hazards of all types of tobacco smoking and helps develop their tobacco cessation counselling skills [29]. Our findings are consistent with research from within and outside the region that recommends integration of education about cigarette smoking and WTS prevention in the health professional students’ curriculum [30–32]. A study from one of the participating institutions had earlier concluded that despite the coverage of the medical curriculum about the hazards of tobacco smoking, students continued to use tobacco and that the curriculum needed to include strategies to help them develop positive attitudes and behaviours to avoid tobacco smoking [33]. Studies from Bahrain encourage efforts to integrate knowledge about the health effects of tobacco smoking in the medical curriculum [8, 32, 33]. Further, a study from the region recently recommended that even dental students, who also have a high level of interaction with their smoking patients, need to be given training on counselling skills so that they may also use their encounters with their patients towards helping them quit tobacco [34]. A previous study on the knowledge of medical students on cigarette smoking hazards reported that although students were knowledgeable about the hazards, they were uncertain about their role in counselling patients to quit tobacco smoking [8].

Our estimate of smoking prevalence (13.5%) was lower than what was previously reported (16.0%) for medical students in Bahrain in 2005 [8]. It is unlikely that the difference is due to the gender composition since the previous study had a similar distribution of both sexes. However, the prevalence of 6.8% of WTS in our study is lower than that reported more than a decade ago for medical students in Bahrain (14.2%) [8]. This is most likely because the previous study had a much larger sample size from only one institution, whereas the current study included medical and nursing students from three institutions.

It is worth noting that this was the first study of its kind in Bahrain involving these three health institutions together in one research project. Participation of students from three health educational institutions in the country through effective collaboration was a strength of this study. In addition, the study has tested tobacco intervention for the first time in the country, therefore; it would help in the development of further interventions towards WTS control. This study has some limitations; firstly, some of the students who participated in the pre-intervention phase did not continue in the intervention and post-intervention survey, which affected the overall response rate. This was probably due to the lack of sufficient motivation among students. Secondly, the investigators considered a matched pair study design with a control group, but it was not feasible in view of the possibility of cross-contamination as well as logistical difficulties. This was the reason that a before and after study design was considered most suitable.

## Conclusion

The intervention improved health professional students’ knowledge of the hazards of WTS. Future studies are encouraged to provide evidence on other effective interventions to prevent and control WTS among youth in Bahrain. In addition, there is a need to review the medical and nursing curricula to emphasize the hazards of WTS and develop prevention strategies in Bahrain and other similar contexts.

## Abbreviations

(AGU): Arabian Gulf University
(RCSI Bahrain): Royal College of Surgeons Ireland-Bahrain
(CHS): College of Health Sciences
(WTS): Waterpipe tobacco smoking
